# Report on Cholera in the Black Sea Fleet

**Published:** 1857-01

**Authors:** B. G. Babington

**Affiliations:** President of the Society.


*Table in connexion with a Report on Cholera in the Black Sea and Baltic Fleets in the Autumn of* 1854.

### BLACK SEA FLEET.

| Name of Ship. | Kind of Vessel. | Name of Surgeon. | No. on board. | Officers. | Seamen. | Stokers. | Marines | Officers attacked | Seamen attacked | Stokers attacked | Marines attacked | Officers died. | Seamen died. | Stokers died. | Marines died. | Situation of ship when attacked. | Date of First Case. | Water used in Cooking and Drinking. | Place and time of Supply of Bread, Milk, Fruit, and Vegetables. |
|---|---|---|---|---|---|---|---|---|---|---|---|---|---|---|---|---|---|---|---|
| Sidon . . | Stm. frigate. | W. R. Dalton. †Charles F. A. Courtney. | 297 | 36 | 213 | — | 48 | 0 | 8 | — | 3 | 0 | 6 | — | 2 | Just arrived at Kustenjeh from Baltschik. | August 13 | Water from Baltschik. | From Baltschik, the day before the first case occurred. |
| Banshee . | Stm. vessel. | None. †James Ross. | 60 | 10 | 28 | 14 | 8 | 0 | 0 | 0 | 1 | 0 | 0 | 0 | 1 | Constantinople. | November 24 | Wells at Constantinople. | Daily from Constantinople. |
| London . | Second rate. | John Douglas, M.D. †A. Irwin. †*Acting* John Coogan. | 812 | 39 | 589 | — | 184 | 1 | 14 | — | 9 | 0 | 6 | — | 6 | Baltschik. | August 4 | Running stream near Baltschik. | From Baltschik during the first nine days of August. |
| Firebrand . | Stm. frigate. | Hugh O'Hagan, M.D. †Edward McSorley. | 194 | 24 | 142 | — | 28 | 0 | 2 | — | 0 | 0 | 2 | — | 0 | Bay of Varna. | August 15 | Constantinople, from a cistern supplied by Belgrade aqueduct. | From Constantinople, where cholera existed at the time. |
| Vulcan . . | Steam troop ship. | James Peters. † None since previous May. | 165 | 22 | 129 | — | 14 | 0 | 1 | — | 0 | 0 | 0 | — | 1 | Constantinople. | September 26 | Distilled water, and water from the shore. | From various places when moving, but chiefly from Constantinople. |
| Niger . . | Stm. corvette. | F. Stupart. †W. V. E. Reynolds. | 170 | 22 | 106 | 22 | 20 | 0 | 1 | 1 | 1 | 0 | 1 | 0 | 1 | Crimea, at the river Alma. | September 23 | Distilled water. | None obtained. |
| Highflyer . | Screw steam ship, 21 guns. | William Kerr, M.D. †Alexander Watson, M.D. | 236 | 29 | 179 | — | 28 | 0 | 1 | 1 | 0 | 0 | 0 | 0 | 0 | Western coast of the Crimea. | September 30 | Distilled water. | At Balaklava. The crew were in daily communication with the shore, where cholera prevailed. |
| Agamemnon | Screw steam ship. | George Mackay, M.D. †Edwin T. Watkins. | 867 | 40 | 595 | Engs. 7 Stkr 33 | 192 | 0 | 12 | 0 | 15 | 0 | 8 | 0 | 14 | Buyukdere Bay. | August 4 | From shore at the Bosphorus, afterwards at Baltschik and Varna, lastly distilled water. | At Buyukdere in the beginning of August, then at Baltschik and Varna. |
| Queen . . | First rate. | John Munro, M.D. †William Telfer. †John Rorie. | 963 | 47 | 720 | — | 196 | 0 | 4 | — | 2 | 0 | 4 | — | 2 | Baltschik. | July 5 | From iron tanks. | In small quantities from Baltschik. |
| Cyclops . . | Stm. frigate. | None. †Charles F. Williams. | 90 | 14 | 68 | — | 8 | 0 | 5 | 0 | 0 | 0 | 2 | — | 0 | Constantinople. | August 16 | From Constantinople. | Bread and milk, but no fruit, from Varna. |
| Megæra . | Iron screw steam troop ship. | James Fisher, M.D. † None. | 155 | 17 | 102 | 22 | 14 | 0 | 3 | 0 | 0 | 0 | 2 | 0 | 0 | Baltschik. | September 6 | Partly spring, partly distilled. | None. |
| Wasp . . | Screw steam sloop. | M. Walling. †George P. Cooke. | 165 | 18 | 129 | — | 18 | 1 | 3 | — | 1 | 1 | 0 | — | 1 | Balaklava. | October (date not given) | Distilled water used, except by those employed in the trenches, who had very bad water. | Free communication with the shore. Supply of vegetables very scanty. |
| Furious . . | Stm. frigate. | R. Fulton, M.D., ill at commencement of cholera; A. Watson, M.D., *Highflyer*, did duty. | 217 | 24 | 165 | — | 28 | 0 | 17 | 7 | 2 | 0 | 10 | 6 | 2 | Baltschik Bay. | August 9 | From the watering place one mile south of Baltschik, good and fine. | Vegetables from Baltschik. Bread scantily supplied. |
| Leander . | Sail. frigate, fourth rate. | Edward Nolloth, M.D. †Frederick W. Blake. †W. J. Baird, M.D. | 500 | 34 | 396 | — | 70 | 1 Boatsw. | 29 | — | 8 | 1 | 15 | — | 5 | Old Fort Bay. | September 20 | Springs of Varna and Baltschik, latter mixed with animal matter, the flocks and herds using the springs. | From Baltschik and Varna, where cholera prevailed among the troops. |
| Vesuvius . | Steam sloop. | John H. Pattinson. †Gilbert King. | 155 | 20 | 115 | — | 20 | 0 | 5 | 1 | 0 | 0 | 3 | 0 | 0 | Off Sulina mouth of Danube; previously off Baltschik. | August 12 | Chiefly from Sulina mouth of the Danube; was sometimes a little brackish. | At Baltschik, from August 5th to 11th, these were procured. |
| Rodney . . | Second rate. | C. R. Kinnear, M.D. †William Duirs, M.D. †Archibald Stevenson. | 789 | 41 | 569 | — | 179 | 0 | 21 | — | 5 | 0 | 6 | — | 2 | Baltschik. | August 10 | Pure water from a spring at Baltschik. | In small quantities from Baltschik. |
| Terrible . | Stm. frigate, fifth class, 21 guns. | Howard R. Banks. †David Henry Wright, M.D. | 300 | 32 7 are engineers. | 183 | 36 | 49 includ. 1 officer. | 0 | 2 | — | 1 | 0 | 1 | — | 0 | Off Cape Loukoul, Crimea. | September 19, 3·30 A.M. | From a small stream at Baltschik. Its qualities stated at large. | All obtained from Baltschik and Varna. |
| Sans Pareil | Screw steam third rate. | James Donovan, M.D. †John T. U. Bremner, M.D. †Richard B. Power. | 630 | 40 | 446 | 22 | 122 | 0 | 13 | — | 12 | 0 | 7 | — | 5 | At anchor at Varna, then at Balaklava, and off the Katchka, Crimea. | July 31 | Distilled water and water from the shore used indiscriminately. | Men could procure all these from the shore at Varna. |
| Retribution | First class fifth rate stm. frigate. | Augustus Slight. †Henry S. Edwardes. | 300 | 20 | 231 | — | 49 | 0 | 1 | — | 1 | 0 | 1 | — | 1 | Cruising in lat. 43·06, long. 38·23. | August 13 | Water taken from a running stream. | Daily obtained from the shore at Kavarnah Bay when the fleet was there. |
| Trafalgar . | First rate. | *Acting* David Lloyd Morgan. †Robert Creighton. | 963 | 39 | 728 | — | 196 | 2 | 92 | — | 31 | 2 | 30 | — | 8 | Anchored off Baltschik. | August 9 | From a stream at Baltschik, running thro' calc. cliffs, disc. from org. matter, used by Bosquet's Div. for washing. | Obtained in abundance from Baltschik. |
| Albion . . | Second rate. | Richard Mason. †G. Mason, M.D. †John F. Pritchard. | 790 | 38 | 581 | — | 171 | 1 | 72 | — | 24 | 0 | 50 | — | 18 | At Baltschik. | August 9 | Spring at Baltschik, a good deal surcharged with lime. | Supplied from Varna and Baltschik. |
| Bellerophon | Third rate. | Dr. Mackay, Mr. Costello; subsequent to cholera, Dr. Carmichael. †Dr. Brown, †Dr. Fisher. | 650 | 40 | 458 | — | 152 | 0 | 9 | — | 6 | 0 | 7 | — | 1 | Varna. | July 31 | Water obtained from Baltschik and Varna. | Had been obtained from Varna daily. |
| Britannia . | First rate. | John Rees. †J. W. Elliott; †H. H. Smith, M.D.; and †A. Irwin. | 1054 | 54 | 782 | — | 218 | 0 | 169 | — | 60 | 0 | 98 | — | 41 | Baltschik. | August 9 | From a rapid stream from the hills a quarter of a mile below Baltschik. | From Baltschik and Varna. |
| Vengeance . | First class second rate. | William Graham, M.D. †John Ward. †William B. Stephens. | 740 | 38 | 523 | — | 179 | 0 | 18 | — | 11 | 0 | 8 | — | 9 | At anchor off Baltschik. | August 8, 9 P.M. | Spring at Baltschik. | From Baltschik. |
| Inflexible . | Paddlewheel steam sloop. | John Watt Reid. †George F. A. Drew. | 195 | 27 | 98 | 23 | 47 | No cholera cases occurred; but diarrhœa was prevalent, and successfully treated with lead and opium pills of the Edinburgh Pharmacopœia, given every half hour till the disease abated. | | | | | | | | Constantinople, Baltschik, Varna, and with the fleet in Aug. & Sept. | No cholera. | Spring water. | Not mentioned. |
| Triton . . | Iron steam vessel. | None. †John Forbes. | 63 | 9 | 48 | — | 6 | No case of cholera occurred. The first case of diarrhœa occurred at Varna; and during the continuance of this diarrhœa, the vessel was at Baltschick and Varna, or at sea with the fleet. Two officers, sixteen seamen, and two marines, were affected. | | | | | | | | At Varna. | First case of diarrhœa Aug. 5 | From a small stream at Baltschik. | Onions only were obtained from Baltschik. No bread or fruit. |
| Spitfire . . | Third class stm. surveying vessel. | Robert Willcox. | 66 | 9 | 50 | — | 7 | No cholera. Thirty-eight cases of diarrhœa were placed on the sick list during the months of July, August, and September. The symptoms did not differ from those ordinarily observed, and they yielded easily to ordinary remedies. | | | | | | | | Position frequently changing. | 38 cases of diarrhœa during July, Aug., Sept. | From Varna Lake, from south side of Varna Bay, and from Baltschik. | Fruit and vegetables disallowed. Bread and milk occasionally from Varna and Baltschik. |
| Spiteful . . | Steam sloop. | Vans C. Clarke, M.D. †Doyle M. Shaw. | 135 | 17 | 99 | — | 19 | | | | | | | | | At anchor near *Leander* at Eupatoria, Oct. & Nov. when *Leander* suffered. | | Brought in casks from Eupatoria, and very good both for cooking and drinking. | Chiefly from Eupatoria. |
| Beagle . . | Steam gun vessel. | †William H. Cameron. | 65 | 10 | 47 | — | 8 | | | | | | | | | Arrived in Black Sea when cholera in squadron had almost ceased. | | Distilled water. | In September at Malta. In September and December at Constantinople. |
| Arrow . . | Despatch gun boat. | †P. W. Govett. | 65 | 8 | 37 | 11 | 9 | | | | | | | | | | | Chiefly distilled water. | At Malta, Constantinople, and Eupatoria. |
| Caradoc . . | Stm. vessel. | †Charles McShane, M.D. | 65 | 11 | 47 | — | 7 | | | | | | | | | | | | |
| Apollo . . | Store ship. | †Richard J. Squire. | 90 | 10 | 70 | — | 10 | In these vessels there was neither cholera nor diarrhœa. | | | | | | | | Baltschik Bay, where the disease was raging between Aug. 11 & 29. | | Water obtained at Malta. | Inferior bread from Baltschik. Fruit not allowed. |
| Fury . . . | Steam sloop. | John Stirling, M.D. †Henry Harkan. | 160 | 22 | 118 | — | 20 | | | | | | | | | | | From a rivulet at Baltschik; also distilled water. | Got from shore occasionally. |
| Modeste . | Sloop. | Alexander Mitchell, M.D. †E. Pearce (not on board). | 141 | 13 | 107 | — | 21 | | | | | | | | | | | | Obtained fresh provisions chiefly from Corfu. |
| Viper . . | Screw steam gun vessel. | None. †T. J. Haran. | 65 | 10 | 47 | — | 8 | | | | | | | | | | | | |
| Total . . . . . . . . . . | | | 12,372 | 884 | 8945 | 190 | 2353 | 5 | 502 | 10 | 193 | 4 | 267 | 6 | 120 | | | | |

### BALTIC SHIPS.

| Name of Ship. | Kind of Vessel. | Name of Surgeon. | No. on board. | Officers. | Seamen. | Stokers. | Marines | Officers attacked | Seamen attacked | Stokers attacked | Marines attacked | Officers died. | Seamen died. | Stokers died. | Marines died. | Situation of ship when attacked. | Date of First Case. | Water used in Cooking and Drinking. | Place and time of Supply of Bread, Milk, Fruit, and Vegetables. |
|---|---|---|---|---|---|---|---|---|---|---|---|---|---|---|---|---|---|---|---|
| Wrangler . | Steam gun vessel. | †William E. O'Brien. | 65 | 9 | 48 | — | 8 | 0 | 1 | — | 1 | 0 | 1 | 0 | 0 | At Woolwich. | August 3 | Pump water at Woolwich. | At Copenhagen during the prevalence of diarrhœa. |
| Hannibal . | Second rate screw steam ship. | John J. Crawford, M.D. †A. R. R. Preston. †C. G. Woolfender. | 620 | 41 27 Fr. | 436 977 Fr. troops. | — | 143 | 0 | 38 | — | 8 | 0 | 15 | — | 1 | At sea in the Baltic, lat. 56 N., long. 5·43 E. | August 1 | Distilled water. | From Copenhagen. |
| Algiers . . | Second rate screw steam ship. | John Andrews. †James S. Ayerst. | 646 460 Rus. prisonrs | 42 | 465 | — | 139 | — | 1 | — | 0 | 0 | 0 | — | 0 | At sea, but previously at anchor in Ledsund. | August 20 | Principally distilled water. | Fresh beef and vegetables were had from transports. |
| Sphinx . . | Sixth rate steam ship. | †John Hickens. | 166 | 25 | 121 | — | 20 | | | | | | | | | In the Baltic. | | No answer. | No answer. |
| Stromboli . | Paddlewheel steam sloop. | F. Negus. †J. S. Adams. | 160 | 21 | 77 Boys 24 | 18 | 20 | Baltic ships not visited by either cholera or diarrhœa. | | | | | | | | In the Baltic. | | From tank vessel at Gottland. | Vegetables from Elsinore, Gottland, *Duke of Wellington*, and *Bulldog*. |
| Gladiator . | Stm. vessel, sixth rate. | D. Hunter, M.D. †D. Porteous, M.D. | 159 | 22 | 108 | — | 29 | | | | | | | | | In the Baltic. | | No answer. | No answer. |
| Valorous . | Stm. frigate, sixth rate. | Stn. Bowden. †William Ray, M.D. | 217 | 23 | 169 | — | 25 | | | | | | | | | In the Baltic. | | No answer. | No answer. |
| Total . . . . . . . . . . . | | | 2033 | 183 | 1448 | 18 | 384 | 0 | 40 | — | 9 | 0 | 16 | 0 | 1 | | | | |

† This mark denotes Assistant-Surgeons.

### TRANSACTIONS

OF THE

### EPIDEMIOLOGICAL SOCIETY OF LONDON.

### Papers and Communications.

### REPORT ON THE CHOLERA WHICH VISITED HER MAJESTY'S BLACK SEA FLEET IN THE AUTUMN OF 1854.

By B. G. BABINGTON, M.D., F.R.S., President of the Society.

ELABORATE and numerous as have been the reports and investigations promulgated from various places in this and other countries with relation to Asiatic Cholera, there yet has remained a source of information freer from the contamination of preconceived notions and prejudices, and furnishing therefore more reliable data than most others for studying the disease, from its first outbreak to its final disappearance. I allude to information supplied by our brethren on board her Majesty's fleets in the Black Sea and in the Baltic, where cholera was so prevalent and so destructive during the autumn of the year 1854.

Every man-of-war has one or more medical officers on board, who are regularly educated members of our profession, and who have all been proved competent by examination, not only under the Royal Colleges of Physicians or Surgeons of the empire, but likewise under the professional head of her Majesty's Naval Medical Service; they are, therefore, as a whole, thoroughly competent medical witnesses and observers, and at least as likely to be impartial as any other members of our body. They are, moreover, under a strict system of discipline; and whatever they are, in the course of their service, called on to do, they do it zealously, no doubt, but still as a matter of duty. In placing, therefore, a series of questions before them, the answers they give are likely to be complete, because they are asked through the medium of authority, which they are bound to obey; and those answers, be it remembered, come from gentlemen who are not obtruding on us views and theories which they are interested in supporting, but simply replying to the best of their abilities to questions which have not originated with or been suggested by themselves. It will be at once perceived that information of this kind, all bearing upon the same points of inquiry, and yet supplied by numerous medical men in isolated positions, and holding no necessary communication with each other, must have a peculiar value; so that, if ever the cause of this mysterious epidemic is to be discovered, it is highly probable that it will be through the medium of some such investigation as that of which this report records the result.

Nor is the nature of the testimony, independent as it is, the only peculiarity in reports from naval medical men. The circumstances with which that testimony is connected are peculiar also. Every ship is an isolated assemblage of persons who are always under the eye of their medical officers, and every event bearing on health or disease being as a matter of duty carefully noted, there is afforded an opportunity rarely met with under any other circumstances, of tracing the origin of all deviations from the healthy condition.

In a town, we inquire with especial minuteness into all the circumstances attending the first two or three cases of cholera. They are the most instructive of any. In a fleet, every individual ship is, as it were, a separate town; and these instances of first cases, accurately noted, are as numerous as are the ships in which cholera has occurred. Again, if we consider the crew or inhabitants among whom the disease appears, we find that they are all placed under nearly the same circumstances; or, if there be any difference, as in the case of officers, as contradistinguished from men before the mast; of marines, as contradistinguished from men who go aloft; of engineers and stokers, who lead a confined life, in contradistinction to other seamen, who are constantly exposed to the air; that difference is definite. We know in what work or occupation every soul on board has been engaged; we know to what vicissitudes of climate they have all been exposed; we know precisely what has been their diet and beverage, what the space which has been allotted to each during the hours of sleep; in short, we know more about them than we can ever hope to know of the inhabitants of any place on shore, how diligently soever medical house to house visitations—so strongly recommended in times of pestilence—may have been carried out. Again, a ship has the great advantage over a town, that its population can be easily moved from place to place, and we therefore have the opportunity of observing whether the cause of cholera exists exclusively in particular veins, currents, or beds of air, or whether it is spread far and wide over a whole region.

It is from these considerations that an examination and analysis of the following reports is undertaken with much interest; and if it be found that they do not give us all the information we seek, we may yet gather from them much that is valuable, and may regard them as affording reliable data for future researches. Lastly, it is under such definite conditions as exist scarcely anywhere but in ships, that fresh endeavours as to treatment can be best carried out; and if any means should prove, on respectable testimony, so successful as to call for further trial under accurate observation, there seems to be no combination of circumstances so favourable for such a trial as is to be met with on board Her Majesty's ships of war.

With these few preliminary remarks, we proceed to give the substance of the Reports from the Black Sea; these being answers to queries which were furnished by the Cholera Committee of our Society. Similar queries were forwarded to Government for transmission to the Baltic fleet, but, except in the case of seven ships which proceeded from the Baltic to the Black Sea, and which consequently received the queries when on the latter station, they never reached their destination; or if they did, the answers have in some inexplicable way disappeared from the offices of the Admiralty and the Board of Health, where every search for them has been made in vain. From the Black Sea, there are altogether 35 sets of answers from the following ships:—

| | | |
|---|---|---|
| 1. Sidon. | 13. Furious. | 25. Inflexible. |
| 2. Banshee. | 14. Leander. | 26. Triton. |
| 3. London. | 15. Vesuvius. | 27. Spitfire. |
| 4. Firebrand. | 16. Rodney. | 28. Spiteful. |
| 5. Vulcan. | 17. Terrible. | 29. Beagle. |
| 6. Niger. | 18. Sans Pareil. | 30. Arrow. |
| 7. Highflyer. | 19. Retribution. | 31. Caradoc. |
| 8. Agamemnon. | 20. Trafalgar. | 32. Apollo. |
| 9. Queen. | 21. Albion. | 33. Fury. |
| 10. Cyclops. | 22. Bellerophon. | 34. Modeste. |
| 11. Megæra. | 23. Britannia. | 35. Viper. |
| 12. Wasp. | 24. Vengeance. | |

Some of these ships had cholera; some had diarrhœa, but no cholera; and some had neither cholera nor diarrhœa; and thus we have been enabled to construct the table which appears at the end of this paper.

The questions which were circulated are as follows :—

1. Name and class of ship.
2. Name of Surgeon.
3. Name of Assistant-Surgeon.
4. No. of Officers.
   „ Seamen.
   „ Marines.
   „ Stokers.
5. Number of cubic feet per individual between decks.
6. Locality and situation of ship when attacked with cholera, and for fourteen days previously.
7. Had bread, milk, fruit, or vegetables been obtained from the shore? If so, when were they obtained, and from what place?
8. Did the crews of boats communicating with the shore, or with shore boats, suffer more, or at an earlier period, than the men who were confined to the ship?
9. Did the disease attack the men of one mess more than in other messes?
10. State the number of officers, seamen, and marines, respectively attacked.
11. The number of fatal cases in each class respectively.
12. Number of cases (if any) without premonitory symptoms.
13. When there were premonitory symptoms, what was their average duration?
14. State the exact date of the occurrence of the first case of cholera or choleraic diarrhœa on board your ship.
15. What appeared to you to be the chief predisposing and exciting causes of the disease?
16. What water was used by the crew for cooking and drinking?
17. If your ship escaped, or suffered but slightly, as compared with others at the same anchorage, or in the same locality at sea, to what do you ascribe such absolute or comparative immunity?
18. Can you state whether the disease had appeared in the Aland Islands previously to the landing of the troops?
19. Can you state whether the disease was more rife in the gulf of Bothnia, or gulf of Finland?
20. Describe the general mode of treatment adopted; and what treatment was most efficacious?
21. Have you any suggestions to offer with respect to prophylactic measures against cholera aboard of ship?

The answers to some of these questions admit of classification under distinct headings, and are given in a table at the end of this paper. There are other answers, however, which do not, readily at least, admit of tabulation, and these we will proceed to consider.

The first of these questions is:—*Did the crews of boats communicating with the shore or with shore boats suffer more or at an earlier period than the men who were confined to the ship?*

This question is answered from the *Sidon*, steam-frigate, in the affirmative: thus—"The boats' crews did suffer more and at an earlier period than the men confined to the ship. The first person attacked was one of the dingy boys, who had been on shore at Baltschik, on the morning of the 12th of August, for sand. He was attacked at 1 o'clock A.M. the following morning."

The *Banshee*, steam-vessel, answers this question by the monosyllable—*no*.

So also the *London*, adding that only three men belonging to boats were attacked at all; but unless we knew how many of her men belonged to boats, we could draw no conclusion from this answer. We only know that the whole ship's company, including officers, amounted to 812 souls, and that of these, 1 officer, 14 seamen, and 5 marines, in all 20 persons, were attacked; which is less than one in forty. If, therefore, there were fewer than 120 men belonging to boats, they had more than their average of attacks among these.

In the *Firebrand*, steam-frigate, the ship's company had leave at Constantinople on the 12th of August, and the only man attacked had been on shore all night and returned intoxicated. It was on the night of the 14th that he fell ill. There was only one other case, that of a boy; who, we are left to conclude, went on shore with the rest, but the date of his attack is not given.

Of the *Vulcan*, steam troop-sloop, the boats' crews were constantly employed at Varna prior to embarking the troops for the Crimea, but the disease did not affect any of them.

In the *Niger*, steam-corvette, no cases occurred among the boats' crews who went on shore in the Danube for fresh meat.

In the *Highflyer*, a large proportion of the men were employed in discharging the cargoes of transports at Balaklava. There were only two cases of cholera in this ship; one was that of a seaman, who had been employed in the working party, and one a stoker, who acted as servant to the Engineer's mess, and had been on shore. Neither of these cases was fatal.

The *Agamemnon* answers *no* to the question; so also the *Queen*, the *Cyclops*, the *Megæra*, the *Wasp*, the *Albion*, the *Britannia*, and the *Trafalgar*; but in this last ship the first case was that of a ship's corporal, who had been on shore on the previous day, and had eaten freely of unripe plums.

The answer from the *Furious*, steam-frigate, is instructive, and shall therefore be given at length.—"Boats' crews did not suffer more than the rest of the ship's company, and they certainly did not take the disease earlier. Of the 12 who were attacked on the first two days, 4 were from boats' crews, and 8 were not. In all 26 took the disease, of whom 11 belonged to boats, and of these 5 died; while of the fifteen others, no fewer than 13 died. As 84 people of the ship's company, which amounted altogether to 217, belonged to boats, 11 will represent pretty nearly the corresponding ratio of the numbers attacked."

From the *Leander*, sailing-frigate, with a crew of 500, the answer is that the first four cases of cholera occurred in men employed on shore at Old Fort Bay, bringing on board flour captured from the enemy; about 60 men were occupied thus on the 19th and 20th of September (the first case occurred on the latter day). The subsequent cases did not fall more on those thus employed than on others remaining on board.

In the *Vesuvius*, steam-sloop, the boats' crews communicating with the shore suffered more, and were nearly the first attacked; while in the *Rodney*, a screw second-rate, the boats' crews are stated to have suffered less and not at an earlier period than the rest of the ship's company.

In the *Terrible*, paddle-wheel steam-frigate, only one case of Asiatic cholera occurred amongst the boats' crews who were in daily communication with the shore at Varna, Baltschik, and the coast of the Crimea, also with the transports, especially those which conveyed horses. In these cholera had prevailed to a great degree during the time they were at anchor off Baltschik and Varna. The man who was attacked was taken ill on the 19th of September, and was the first case in the ship. The previous day he had been employed in landing horses at Loukoul. The second case of cholera occurred on the 22nd of September, in a delicate lad who had been suffering from bilious diarrhœa for two days, but had concealed it. He fell rapidly into a state of collapse, from which he was with difficulty extricated; consecutive fever set in on the 25th, which terminated fatally on the 30th. He had had no communication with the shore for many months.

The answer from the *Sans Pareil*, third-rate, is that nearly all the ship's company formed part of the different working parties on shore, so that no conclusion could be come to.

From the *Retribution*, steam-frigate, we have the answer that, from the number of seamen during this period on the sick list, suffering from diarrhœa, and who were attached to the different boats of the ship, it is probable, that these men did suffer more than those who were confined on board, that is from diarrhœa and choleraic diarrhœa.

With respect to the *Bellerophon*, constant intercourse with the shore was kept up by all on board, so that no inference could be drawn.

The answer from the *Vengeance*, first-class second-rate, is, that out of 18 seamen attacked, 4 only belonged to boats, and that these were not the first cases.

It thus appears that in seven ships those having communication with the shore were first affected, the notion being in so far favoured, that the disease was caught by contagion; while in fourteen cases the disease did not appear first in those who were in communication with the shore, and in two cases there was no evidence either on the one side or the other.

The following are answers to the question as to *the numbers of cases (if any) without promonitory symptoms.*

*Sidon.*—One case without premonitory symptoms, the other ten with premonitory symptoms, lasting from 12 to 48 hours.

*Banshee.*—None without. Only one man attacked, who had smart diarrhœa for 8 hours.

*London.*—None without. Duration of premonitory symptoms, a few hours to 3 days.

*Firebrand.*—In the two cases, the one for 8 hours, the other for 2 or 3 days.

*Vulcan.*—None without. Diarrhœa for about 12 hours.

*Niger.*—All three had diarrhœa from 1 to 3 days.

*Highflyer.*—None without. In the only two cases the one was purged 9 hours.

*Agamemnon.*—None without. Premonitory symptoms from 2 to 6 hours.

*Queen.*—One uncertain. Premonitory symptoms about 2 days.

*Cyclops.*—Without premonitory symptoms two; with premonitory symptoms three; which lasted 3 hours.

*Megæra.*—One without premonitory symptoms, two with; lasted 2 days.

*Wasp.*—None without.

*Furious.*—Twenty-six attacked. Cannot tell, not being with the ship.

*Leander.*—None without. Thirty-eight cases. Premonitory symptoms 2 or 3 days. Diarrhœa in the ship more or less for 3 weeks.

*Vesuvius.*—Without premonitory symptoms, one seaman; in the rest the premonitory symptoms lasted about 24 hours.

*Rodney.*—None without. All had premonitory symptoms from 2 or 3 days to 12 hours.

*Terrible.*—One case without. One with bilious diarrhœa for 2 days.

*Sanspareil.*—Uncertain evidence.

*Retribution.*—Two or three cases without premonitory symptoms; where existing they lasted from a few hours to a day.

*Trafalgar.*—Doubtful whether any were without premonitory symptoms.

*Albion.*—In eight cases out of ninety-seven, no premonitory symptoms.

*Bellerophon.*—Three cases out of eighteen occurred without diarrhœa; duration of premonitory symptoms, 6 hours to a week.

*Britannia.*—No premonitory symptoms in half the cases.

*Vengeance.*—Three out of twenty-nine without; where there were premonitory symptoms, they lasted from 2 to 3 days.

It thus appears that out of 711, which was the number of attacks of cholera in the whole Black Sea fleet from which returns were received, there were, without premonitory symptoms in the—*Sidon,* 1; *Cyclops,* 2; *Megæra,* 1; *Vesuvius,* 1; *Terrible,* 1; *Retribution,* 3; *Albion,* 8; *Bellerophon,* 3; *Britannia,* 114; in all 134 cases, or more than one in six.

In the *Britannia,* where there were altogether 229 persons attacked, the answer, probably from the surgeon, Mr. Rees, is thus given: "Not able to state with accuracy, but would give as my opinion, that one half at least had no premonitory symptoms whatever."

We now come to the opinions of the medical officers on *the chief predisposing and exciting causes of the outbreak of cholera in the fleet, the comparative suffering of each ship,* and *the treatment and suggestions as to preventive measures.*

The medical officer of the *Sidon,* Mr. Dalton, considers that some peculiar and subtle poison in the atmosphere was the exciting cause. The excessively sultry and oppressive weather with the thermometer between 80 and 84, and the barometer between 29·93 and 30·34, was, in his opinion, the predisposing cause. The *Sidon's* comparative immunity may have arisen from her not having remained so long as other ships at anchor at Baltschik and Varna, where the cholera raged, and from her having put to sea for three or four days after the first case occurred. The treatment at the commencement was half a drachm of calomel, or calomel and rhubarb; subsequently two grains of calomel and a quarter of a grain of opium, every two hours, with stimulants. In collapse, warm baths, turpentine epithems, mustard poultice to the stomach, hot bricks to the extremities, and friction and heated sand-bags between the thighs were employed. To allay vomiting, chloroform was given in ten-drop doses. During convalescence, tonics and stimulant beverages were used. As preventive measures, Mr. Dalton suggests—ventilation, cleanliness, chloride of zinc as a disinfectant, recreation and amusement among the men, with generous diet and fresh provisions. He also recommends quinine as a preventive, and observes that the officers, living better, were more exempt than the men, and concludes, therefore, that an impoverished state of blood predisposes to the complaint.

The medical officer of the *Banshee*, Mr. Ross, considers that there were no discoverable predisposing or exciting causes; and he offers no observations as to the comparative immunity in that vessel. In the only fatal case that occurred, acetate of lead and opium in large doses, and other powerful astringents, scruple doses of calomel, and blisters, and stimulants, were all used without effect. He suggests as preventives—thorough ventilation, avoidance of damp, generous living, abstinence from green and unripe fruits, and a careful attention to the state of the bowels.

Dr. Douglas, of the *London*, is unable to say anything about predisposing or exciting causes. He thinks it possible that the ship suffered but slightly, from having been kept clean, dry, and well ventilated. The crew having been guarded as much as possible from the heat of the sun, and the ship having put to sea early. The diarrhœa was treated with strong astringents and opium; the cramps and vomiting with large doses of camphor, calomel, and opium, sinapisms to the epigastrium, and stimulating frictions to the extremities. He recommends as preventive measures—avoidance of communication with infected places and ships, cleanliness, ventilation, and dryness of decks, good food, warm clothing, avoidance of excesses, and of exposure to night air, wet, and hot sun.

In the *Firebrand*, of two fatal cases that occurred, one was exposed to predisposing causes on shore. The comparative immunity is attributed, by Dr. O'Hagan, to the frequent shifting of the ship's place, the full occupation of the crew, and the strict adoption of precautionary measures and cleanliness. One case was treated with sulphuric acid, the other with ether, opium, brandy, warm bath, and warm frictions: both died. The doctor suggests, as cholera rarely appears without premonitory diarrhœa, the establishment of a strict police to watch the food introduced, to enforce cleanliness and ventilation, and to report all cases of diarrhœa.

In the *Vulcan* there was only one case. The man drank a quantity of cider, and neglected the premonitory symptoms. The treatment consisted in a full dose of calomel, followed by smaller doses of calomel every two hours, and external frictions; but collapse came on and death ensued. Mr. Peters thinks that the usual measures adopted in well regulated ships to keep men in health are likely to be beneficial, viz.: to cleanse the bottoms of the ships, to avoid exposing the men early in the morning without food, to enjoin the free use of flannel, and to feed the crew on fresh meat if procurable.

In the *Niger*, the three men attacked, of whom two died, all belonged to a party of fifty employed from September 21 to 26, in bringing down wounded men and cholera cases from the Alma. The treatment pursued was calomel in large doses, opium, mustard sinapisms, stimulating liniments, hot baths, friction, and brandy.

In the *Highflyer*, there were no fatal cases; the predisposing causes, enumerated by Dr. Kerr, are—fatigue, exposure to the sun by day, and cold at night. The immunity of the ship is attributed to her having remained but two days at Baltschik, and then having gone to Constantinople, which was healthy; also to a good ventilation of the decks. The treatment adopted was, by opiates, astringents, and stimulants, external heat, counter-irritants, and frictions. Dr. Kerr considers the avoidance of exposure to fatigue in the sun, and to sudden chill afterwards, as also the use of proper diet, to be powerful means of warding off the disease.

In the *Agamemnon*, there were 27 attacks, and 22 deaths. Dr. Mackay considers the predisposing causes to have been intemperance and a relaxed state of bowels; the exciting causes a peculiar poison in the atmosphere rendered contagious by the evacuations from the Nile, and foul or confined air in badly ventilated spaces between decks, or in the hold. He remarks that those only were seized who worked in transports, which had either suffered from cholera or were very dirty and foul. The prophylactic measures adopted were the giving drachm doses of quinine wine to watering parties and to those who had been at work in suspected ships, and the use of chloride of zinc in the sick bay and patients' bedding. The remedies adopted in the epidemic diarrhœa were chalk mixture with tincture of catechu, and of opium, and acetate of lead with opium. In cholera, as well in the first stage as in collapse, calomel and opium were exhibited either in large or in frequently repeated doses. Carbonate of ammonia was tried, and also castor oil. No remedy had any decided effect. Oft repeated two-grain doses of calomel were considered the most successful means, and these were occasionally combined with opium, while chloroform, sulphuric ether and other diffusible stimulants were used according to circumstances, and frictions, hot bottles, etc., were employed externally to relieve cramp and restore heat. The prophylactic measures suggested, are — all possible ventilation, the free use of chloride of zinc, and a change of the locality by proceeding to sea for a cruise. The administration of quinine wine to those going on board suspected ships or on watering duties to the shore is recommended.

In the *Queen*, the six persons attacked, all died; and there were also three who died of diarrhœa. No suggestions are offered respecting either predisposing or exciting causes. The treatment was by stimulants, opiates, turpentine frictions, carminatives, and hot water tins to the stomach. In the diarrhœa, acetate of lead and opium, chalk mixture, and other astringents, were given. The prophylaxis recommended is—to attend early to the diarrhœa by the use of mild astringents, and afterwards to exhibit compound tincture of rhubarb with laudanum.

In the *Cyclops*, there were five attacked, of whom two died. Mr. F. Williams considers the predisposing cause to be a general cachectic state of constitution. The slight visitation of his vessel he attributes to free ventilation and cleanliness. His treatment in those that recovered was, saline, as recommended by Dr. Stevens, with hot sand and hot blankets externally; large doses of calomel were added to the saline treatment in those who died. His recommendations with regard to prophylaxis, are—free ventilation and cleanliness, combined with dryness, and the use of chloride of zinc.

In the *Megæra*, Dr. Fisher reports that three were attacked and two died. In his opinion, the ordinary predisposing causes of all epidemic diseases are also those of cholera. He attributes the nearly absolute immunity of his ship to the abundance of space and the free ventilation. As to treatment, he only states generally, that it was stimulating internally and externally. His prophylaxis is the immediate treatment of diarrhœa, however slight, and a sedulous attention to cleanliness and ventilation.

In the *Wasp*, there were five attacked, of whom two died. Mr. Walling considers the predisposing causes to have been exposure to wet, cold, and wind, with bad food and clothing. In his opinion all lowering causes predispose to the disease.

With reference to the exciting cause, he remarks, that all the cases occurred among those who served with the naval brigade. His treatment was to diminish the quantity of fluids taken, to apply hot water externally, at the same time preventing its evaporation, and to exhibit internally calomel and camphor, in doses of two grains each, every hour, having previously given a large dose of calomel and opium. His prophylaxis is—good clothing and feeding, with comforts, a clean ship and change of place.

In the *Furious*, 26 were attacked and 18 died. Dr. Fulton having been taken ill, Dr. Alexander Walton took charge of the ship, but not till six days after the last case occurred. He could not therefore give any account of either the causes or the treatment of the cases. The prophylactic measures, recommended by him, are cleanliness, dryness, and ventilation of the ship, with good clothing for the men, the avoidance of long fasts and fatigue, and immediate attention to all cases of diarrhœa.

In the *Leander*, there were 38 attacked, of whom 21 died. The only cause, according to Mr. Nolloth, the surgeon, was atmospheric influence. His treatment seems to have been solely external; at least, he only mentions heat and friction, mustard poultices, and turpentine epithems. In choleraic diarrhœa, he found most benefit from turpentine and tincture of opium, but states that dilute sulphuric acid was also much employed. He has nothing to recommend as prophylactic beyond general hygienic measures, and especially dryness of the ship.

In the *Vesuvius*, there were three persons attacked, of whom one died. Mr. Patterson considers the disease to have been excited by some atmospheric morbific cause; while as predisposing causes, he mentions the immoderate use of fruit, and exposure to heat. His treatment was to give a dose of from ten to fifteen grains of calomel, followed by two-grain doses every two or three hours, sometimes with small doses of opium, hot flannels with oil of turpentine to be applied to the epigastrium, and friction to be employed for cramps. As prophylactic measures, he recommends that the use of salt provisions should be suspended, and fresh meat with vegetables in moderation substituted; that fruit should be avoided, and that strict attention should be paid to cleanliness, dryness and ventilation. He likewise enjoins change of place on the outbreak of cholera.

In the *Rodney*, of 26 attacked, 8 died. Dr. Kinnear considers the exciting cause to have been atmospherical, the predisposing cause, bad food, giving rise to diarrhœa. His treatment was astringent mixtures with lead and opium for diarrhœa. In collapse, friction and warmth, and all means calculated to restore circulation. He states that creasote, hydrocyanic acid, and saline treatment were all tried with doubtful effect. In the way of prophylaxis, nothing was done beyond the adoption of the usual hygienic measures; no vegetables, cheese, or irritating articles of food ought to be allowed, and diarrhœa should be treated immediately.

In the *Terrible*, there were three attacked, of whom one died. Mr. Banks considers the exciting cause to have been atmospherical. On the 14th of August, when the first case of choleraic diarrhœa occurred, he states that a hot blast came off from the hills about Baltschik, and continued to blow nearly half an hour, after which the air became many degrees cooler. It passed over the war steamers *Terrible* and *Fury* and over a fleet of 42 transports, all at anchor. Several rapidly fatal cases of cholera occurred in the transports, in which the drainage from the horses had made its way through the shingle to the hold, so that these vessels could not be purified. Previous to their arrival from the Bosphorus nearly all their crews had suffered from diarrhœa, dysentery and typhoid fever, which predisposed them to attacks of cholera. The town of Baltschik was healthy; but just over the anchorage, eight thousand French troops, who had lately returned from the Dobrudscha, and had suffered severely from cholera, were encamped. That the ship's company in the *Terrible*, among whom bilious diarrhœa had been almost universal, suffered so slightly from cholera, is ascribed by Mr. Banks to the excellent state of the men's health, and their regular habits; to the ship having been constantly on the move, and to the crews having had no communication with places on shore where they could commit excesses. They were thus better able from constitution to resist the choleraic poison. The same immunity from cholera was enjoyed by this ship when last in commission, in August and September, 1850. Cholera then was raging at Malta, and in the squadron which had arrived in July from Salamis Bay. The crews of other ships were allowed to visit the shore, where the usual excesses were indulged in. The crew of this ship had not been on shore for more than six months, and were in robust health, and able to resist cholera although they suffered from diarrhœa. The treatment found successful in diarrhœa with coldness of abdomen and flatulence, was as follows: tincture of opium, spirits of turpentine, of each thirty drops; spirit of camphor, ten drops; peppermint water one and a half ounce. Seldom more than two doses were required. The patient was kept in bed, and sinapisms were applied over the abdomen in the event of his suffering from tormina. Arrow root and brandy were employed for sustenance. In decided cholera, calomel and opium freely given did good with some, bringing the patient out of collapse, but with a sequela of fever; with others, these remedies did no good. Under a rapid loss of vital power the stimulating plan both internally and externally is recommended. As prophylactic suggestions—the mess deck is to be kept as dry as possible; when it is washed, hot water should be used, and the men not allowed to go on it till it is quite dry. When the men are turned out early, they should have a ration of tea or coffee before going on deck, in addition to their breakfasts, for diarrhœa has appeared more frequently between four and seven A.M., than at any other time. Provisions should be thoroughly cooked; some men are stated to prefer salt pork unboiled, but that should be forbidden; all fruit brought on board should be examined, and that which is unripe or decayed rejected; above all, the men should be enjoined to apply for medical aid on the least appearance of bowel complaint.

In the *Sans Pareil*, there were 25 attacked, of whom died 12. Dr. Donovan is not aware of any exciting cause except the prevalence of cholera at Varna and among the transports. The different ships suffered differently at the same anchorage without any apparent cause, if the internal management of the ship and cleanliness be excepted. Dr. Donovan's treatment was by heat and friction externally, and by stimulants of all kinds internally. Dilute sulphuric acid was also employed to allay sickness and diarrhœa, as also calomel in small doses; but when the collapse was sudden or occurred early, nothing seemed to avail. The only prophylactic in his opinion is, to keep the body in the best state of health.

In the *Retribution*, 2 were attacked, and both died. The predisposing causes, according to Mr. Slight's opinion, were high temperature, drinking cold water when hot, exposure to cold damp air, and the excessive use of fruits and vegetables. He offers no remarks on the exciting cause. In the treatment, calomel, confection of opium, and quinine, with all means for stimulating the circulation and bringing about reaction, were employed in the cholera cases. The prophylactic measures recommended are—ventilation, cleanliness, the prohibition of fruits, green vegetables, and cold water; protection of the body by flannel, avoidance of high temperature by day and cold at night, fumigation of the hold, whitewashing and the free employment of chloride of lime; also the immediate treatment of premonitory symptoms.

In the *Trafalgar*, 125 men were attacked, and 40 died. Mr. Morgan could not trace out any predisposing cause. The finest men were attacked, and the weak were spared.

The only exciting cause which Mr. Morgan could surmise was the use of water from Baltschik, which was impure in consequence of having been used for washing linen, etc., by Bosquet's light division, just returned from the Dobrutscha. The fact that this ship had fewer deaths, in proportion to the attacks, than others, Mr. Morgan attributes to her having quitted the station and got further from land than any other ship; to the allowing a full circulation of air, to the cleansing by chloride of zinc; to the use of quinine wine to every man each morning; and to the wearing of warm clothing. Of the treatment, which was various, the most efficacious was Dr. Billing's, *i. e.*, two grains of tartarised antimony, and half an ounce of sulphate of magnesia, in half-a-pint of water; of which mixture a table spoonful was taken every half hour. The prophylaxis recommended is—free ventilation, cleansing the ship with chloride of zinc, and keeping the decks dry by swinging stoves.

In the *Albion* there were 97 cases of attack, of which 68 died. Mr. Mason observes that he is unable to assign any cause for the sudden and almost simultaneous outbreak of the disease in so many ships of the squadron. There was no peculiarity observable in the condition of the atmosphere prior to the outbreak, but the disease had been prevailing for three weeks within twenty miles of the ship. All the cases were treated upon the same plan, which was to give a scruple of calomel, with a grain and a half of opium, at the commencement, and to continue the calomel in ten grain doses every hour until the rice water discharges ceased, and the alvine evacuations assumed a feculent character; frictions, the application of hot water, and, in several cases, stimulants, were used without, however, it appearing that they were of any benefit. Mr. Mason thinks that when cholera is known to prevail in the neighbourhood of the port where a ship is lying, it is desirable that the ship should put to sea, and that the distance she proceeds should be such as to give a reasonable hope of her getting clear of the epidemic influence. The men should have warm clothing, good fresh meat and vegetables, and an ounce of quinine wine each before breakfast.

In the *Bellerophon,* there were attacked 16, of whom 8 died. Dr. Mackay considers the exciting cause to have been an epidemic influence, to which the men were predisposed from having been hard worked and exposed to much vicissitude of weather. Fruits were prohibited. The men-of-war at Varna suffered less than those at Baltschik, but for no apparent reason. The treatment was by stimulants of ether, ammonia, and opium; champagne and brandy and water, or ale, for drink, with frictions, hot fomentations, and sinapisms. Calomel was also given in one or two cases in large doses. By these means the purging and vomiting were arrested; but in some a comatose state supervened, from which none recovered. Dr. Mackay's suggestions as to prophylaxis are cleanliness, ventilation, and dryness of decks and clothing, the prohibition of unripe fruit, and a prompt attention to diarrhœa. He remarks that non-communication appears to possess no influence.

In the *Britannia* there were attacked 229, of whom died 139. The crew were in a high state of health when the disease broke out, so that Mr. Rees cannot assign any exciting or predisposing cause for its advent. The great outbreak, however, he thinks, was probably caused in a great degree by the closing of the deck ports on the previous night, on account of the boisterous state of the weather. The treatment was various: sulphuric acid, calomel and opium in large doses, calomel, lead, and opium, chloroform, creasote, various stimulants internal and external, etc. All means signally failed during the advance and at the climax of the disease, but during its decline medicines acted beneficially; and then a combination of lead, opium, and calomel was of most use. The prophylaxis recommended is the keeping the men in a high state of health, and putting to sea in favourable weather. This measure proved useful in the majority of cases, and its want of success in the *Britannia* was owing to the misfortune of being obliged, from the weather, to close the ports when the disease was at its height.

In the *Vengeance* there were attacked 29, of whom died 17. The predisposing cause, according to Dr. Graham, was heat of weather; the exciting cause atmospheric influence. This ship suffered moderately. The treatment consisted in the application of external warmth, and the internal administration of calomel and opium, but not to a great extent; stimulants were also used, but no special mode of treatment is recommended. The prophylaxis suggested is—dryness, cleanliness and ventilation, good food, and the removal of the ship from a diseased locality.

The *Inflexible*, paddle-wheel steam sloop, with a crew, including all hands, of 195, had no cholera, although she was at Constantinople, Baltschik, Varna, and cruising with the fleet in August and September 1854, when cholera prevailed. Diarrhœa was however very prevalent; how many cases occurred is not stated. It was successfully treated in all by the lead and opium pill of the Edinburgh Pharmacopœia, given every half-hour or hour until the disease was checked, means being adopted to prevent the men from eating unripe fruit and other injurious articles of diet. The surgeon of this ship, Mr. John Watt Reid, treated cases of cholera from the *Britannia*, on board the *Apollo*, in August 1854, in the following manner:—Lead and opium pills after each motion, or alternately with chalk mixture. Chloroform, from ten to twenty-five minims, was given, not in extreme collapse, but in some cases where the cramps were severe, the vomiting urgent, and hiccup troublesome. When kept down, as it frequently was, it generally seemed to give relief, and certainly did so in a number of instances. Frictions and heat were used for cramps, sinapisms to the epigastrium for hiccup, and also effervescing draughts. In extreme cases, brandy and wine were given. The immunity of the *Inflexible* from cholera is attributed to the care that was taken of the men on duty, the early application of treatment to diarrhœa, and the means adopted to prevent the use of unripe fruit and injurious articles of diet. No suggestions are offered regarding prophylaxis.

The *Triton* war steam vessel, with a crew of, all hands, 65, had no cholera on board, but 20 cases of diarrhœa, none of which proved fatal. The ship was at Varna when the first case occurred, and during the continuance of diarrhœa was at Baltschik and Varna, or at sea with the fleet. On the whole they had neither frequent nor intimate communication with the shore, with shore boats, or with other vessels of the squadron. The average duration of the diarrhœa cases was seven days. The first case occurred on the 5th of August. As for predisposing causes of diarrhœa, there were none recognised by Mr. Forbes. The crew was in good health, and there was no change in their habits, duties, or diet, to account for the outbreak. With regard to the state of the atmosphere, the days were hot and sultry, and the night air remarkably moist and chilly. The milder cases were treated with chalk mixture and opiate confection. Those with vomiting and occasional cramps had, with good effect, pills of five grains of calomel and a grain of opium, repeated according to circumstances; and when purging continued, after the cessation of pain, pills of five grains of acetate of lead and half a grain of opium were given three or four times a day, according to the urgency of symptoms.

The *Spitfire*, with a crew of, all hands, 65, a third class steam surveying vessel, had no cholera, although communication with the shore at Varna and Baltschik was uninterrupted. She had diarrhœa, however, during the months of July, August and September 1854, and the boats' crews suffered most, from the fruit and other things which they, more than the rest of the ship's company, had the opportunity of indulging in on shore. There were 38 cases of diarrhœa in all placed on the sick list, and the symptoms did not differ from those ordinarily observed. The exciting cause seemed to be some peculiar atmospheric agency. The predisposing causes were exposure to wet and night air, and errors in diet, especially in eating unripe fruit. The escape of the ship from cholera is attributed by Mr. Wilcox to its frequent change of position, so as not to have remained long in the same current of air; and to the facility in a small vessel of observing the state of men's health, their mode of living, and the first appearance or indication of the disease. The diarrhœa is stated to have yielded to ordinary remedies; and the only prophylactic suggestions recommended are, besides cleanliness and ventilation, a strict observance of the crew that they may obtain medical treatment on the first appearance of symptoms.

The *Spiteful* steam sloop, with 135 of all ranks on board, was free from cholera; yet she used the same water, obtained from Eupatoria, as the *Leander*, and was for a week in October and three weeks in November, 1854, lying close to her, during which periods she was suffering severely from cholera. Why the one ship suffered while the other escaped is not considered explicable by Dr. Clarke: and there are no special suggestions recommended by him for the prevention of the disease, beyond cleanliness, a strict surveillance over the shore boats, so that they may not be the means of introducing into the ship unwholesome food or unripe fruits, the use of flannel belts round the abdomen; and lastly, a sufficient supply of clean bedding—a point often altogether overlooked on board ship.

The *Beagle* steam gun vessel, with 65 souls on board, was never attacked with cholera or choleraic diarrhœa. She used distilled water, but her immunity is attributed to her late arrival in the Black Sea, when the cholera had all but ceased, and to her being continually on the move.

The *Arrow,* a despatch gun boat, with 65 souls on board, was never attacked. She used distilled water, and Mr. Govett, her surgeon, makes it a question whether to this cause is to be attributed her immunity. He suggests as preventives, in addition to cleanliness and dryness, the free use of chloride of zinc, the closing the scuttles and ports at night as much as possible, and the use of distilled water.

The *Caradoc* steam vessel, with 65 souls on board, had no case of cholera. No remarks whatever are offered by Mr. Charles McShane, her medical officer.

The *Apollo* store ship had 90 souls on board. She was not attacked with cholera, although lying in Baltschik Bay, where the disease was raging between the 11th and the 29th of August 1854. Inferior bread was daily brought by a boat from Baltschik; but the fruit, not being ripe, was not allowed to enter the ship. The water in use was obtained at Malta. Several of the merchant ships lying close to the *Apollo* suffered; and, moreover, she received the men of the *Britannia,* who had been attacked at sea, amounting in number to 89; 26 of whom died. The ship's company occupied the lower deck, while the sick men from the *Britannia* were on board. It seems very extraordinary that this ship should have escaped. Both contagionists and non-contagionists would find abundant reason why she should have been attacked.

The *Fury* steam ship had 160 souls aboard. She was not attacked by cholera. Her water was partly obtained from the rivulet at Baltschik, and partly from condensed steam. Dr. Stirling, the surgeon, can offer no opinion as to the cause of immunity; for the vessel was surrounded by ships that were attacked, communication with which, as well as with the shore, was freely permitted and actually took place; and the crew devoured unripe plums, pears, apples, etc. without any stint. There were, however, no means of getting spirits.

The *Lynx* steam vessel, the number of whose crew is not stated, had no cholera; and her surgeon, Mr. Johnstone, offers no remarks whatever.

The *Modeste* sloop had 141 souls on board. She has had no cholera since she has been in commission. She was employed fourteen months on the Ionian station, during which time she had a free allowance of fresh provisions, obtained chiefly from Corfu. There was no cholera in the Ionian Islands. No special suggestions are offered by Dr. Mitchell with regard to preventive means.

The *Viper* screw steam gun vessel had 65 souls on board. She did not suffer from cholera, and no remarks in elucidation of this fact are offered by Mr. Haran.

The following are the Baltic ships which had cholera :—

The *Wrangler*, a steam gun vessel, with 65 souls on board, had an assistant surgeon, Mr. W. E. O'Brien. He answers the question respecting the comparative suffering of boats' crews in the negative, and had no cases without premonitory symptoms. He had only two cases of cholera; of these one occurred at Woolwich, on board the hulk, on the 3rd of August, 1854, and the other on board the *Wrangler*, which took place on the 1st of September. The habits, occupation, and manner of living of the ship's crew are stated to have been so opposite in those attacked, that no opinion could be formed as to the predisposing causes; but the exciting cause which appeared most common was sudden change of temperature. The almost complete immunity of this ship from cholera is attributed to her having arrived in the Aland Sea after the disease had nearly subsided in the fleet. The whole of the ship's company, however, had diarrhœa of some kind previously; and there was no instance of a second attack. The treatment of this diarrhœa was by sedatives, antacids, and astringents. Where symptoms of collapse set in, calomel and morphia, given every hour, with sinapisms to the epigastrium, were found the most effectual remedies; and a large number of cupping glasses applied over the limbs and body, and frequently moved, are stated to have had a good effect in restoring warmth and preventing cramp. The chief suggestion as to prophylactic measures is that the bilges be kept clean. In screw steamers much tallow and oil drop into the bilge, and together with refuse matter form a solid mass on the surface of the wood, which is impervious to chloride of zinc. It is therefore recommended to line the bilges with metal, and having let the water from the boilers with caustic alkali in it run into them, to pump them out before using the chloride of zinc.

The *Hannibal*, a second class screw line-of-battle-ship, with a ship's company of 620 men, together with 977 French troops, was attacked with cholera in lat. 56 N. and long. 5·43 E., two days after arrival at Ledsund, near Bomarsund. She had 38 seamen and 8 marines attacked with malignant cholera, but no officers, while there were 232 cases of choleraic diarrhœa, and among them nearly all the officers. These cases were exclusive of those which occurred among the French troops, of whom many had the same affection.

Fifteen seamen and one marine died of cholera; but of these, two cases were relapses during perfect convalescence, brought on from irregularity in diet, and five of them died of secondary fever. There was, however, no death among those affected with choleraic diarrhœa. Of the French soldiers, two died on board; but about 40 died the day after landing. About six or eight of the cholera cases were without premonitory symptoms; but where these existed, their average duration was between twenty-four and forty-eight hours, though in some cases not more than six hours. The first case of choleraic diarrhœa occurred on the 19th of July 1854, and of cholera on the 1st of August 1854. It was that of a French soldier; and the following day one of the ship's company was attacked, and died in a few hours. The predisposing and exciting causes of the disease were as follows:—Too crowded a state of the ship (upwards of 1,500 individuals being on board); insufficient ventilation, arising from the fittings necessary for the troops and the necessity of shutting the lower deck ports at night; change of diet and mode of living, nearly every one on board having been unaccustomed to a sea life; insufficient clothing; and the use of the water distilled on board, which was at first mixed with salt water, in consequence of the same hose having been employed for pumping both kinds of water. From these causes, and perhaps also because the crew consisted of very young and unseasoned men, the *Hannibal* suffered more than any other vessel in the Baltic, except some of the French line-of-battle ships. From information derived from the Russian officers, prisoners of war, it appeared that no case of cholera had ever occurred in the Aland Islands previously to the landing of the French troops. The treatment of the cholera cases was, externally, heat and stimulating frictions to the surface and mustard plasters to the epigastrium and legs; internally, a scruple of calomel with half a grain of opium immediately, followed by two grains of calomel every hour afterwards until mercurial fœtor was observable; drachm doses of spirit of nitric ether occasionally; effervescing draughts of carbonate of soda and citric acid every five or ten minutes; cold water, tea, and lime juice, as much as the patient chose. The calomel and the friction allayed the cramps almost instantaneously, and the effervescing draughts were found the most efficacious means and the most agreeable for relieving sickness. Dr. Crawford considers opium and brandy worse than useless. Under the above treatment 16 cases only out of 46 of true blue Asiatic cholera, all in complete collapse, died. No case of cholera occurred on board the *Hannibal,* between the 14th and 19th of August. On the latter day about 350 Russian prisoners came on board, for a passage to England, from an infected French steamer. Two of them were immediately seized with cholera. Diarrhœa was also prevalent among them; it was attributed to their having drunk salt water on board the steamer, not being able to procure fresh. Between the 19th of August and the 1st of September, 50 of them had malignant cholera, and 19 died, two of typhoid pneumonia after recovery from cholera, and six of secondary fever. The treatment was the same as that already stated. Several of the Russians who died were between 45 and 50 years of age; some were pensioners and several were convicts. Dr. Crawford states that he gave these men as much vinegar and water as they chose to drink, as they were accustomed to sour diet; and he is inclined to think that such a beverage would be beneficial in all cases of cholera. Acetic acid he considers to be, when combined with opium, a powerful remedy in diarrhœa.

Very few persons in the ship escaped diarrhœa; 232 were placed on the sick list as the most severe cases, and were certainly as truly *cholera* as most of the cases returned under that head in Hospital Reports. Very many besides were treated without being put upon the sick list. The French did not suffer so generally from diarrhœa as the English, which may perhaps be explained by their better clothing, by their having no fatigue to undergo, no exposure to the weather or night air, and to their not drinking so much water as our men who had much work to do. The diarrhœa was treated at first with large doses of calomel, afterwards with two grains of calomel and half a grain of opium every two hours, together with chalk mixture, tinctures of opium and of catechu, and other astringents. Of all remedies, the most effectual was calomel and opium with chalk mixture. Not a single case died of this disease. Dr. Crawford suggests, that during the prevalence of cholera, the men should not be overworked, they should be allowed a fair quantity of sleep, and not be obliged to leave their beds too early in the morning; that they should not be exposed unnecessarily to wet or cold or night air; that they should have their meals at regular hours, and be allowed sufficient time for them; that their clothing should be warm, and their bedding aired occasionally. With regard to the ship itself, it should be well ventilated, the decks kept clean and dry, and the hold frequently washed with chloride of zinc. All depressing influences should be avoided, and amusements, as dancing and singing, encouraged.

The *Algiers,* second-rate screw steam ship, had a crew, taken altogether, of 646 men, and in addition to these 460 Russian prisoners. None of the boats' crews was attacked with cholera, and only one case occurred among the ship's company, which case did not prove fatal. One Russian prisoner was attacked and died, and he had no premonitory symptoms. The first case occurred on the 20th of August, 1854. The disease was raging epidemically among the French men-of-war when the *Algiers* arrived at Ledsund. Among the English ships it was on the wane, only a case or two appearing now and then. The first case appeared on the day on which the Russian prisoners were received; one of these was attacked during the night and died the next day. These prisoners came from a French hospital-ship, in which the disease was very rife. After this, diarrhœa of a choleraic character appeared among the prisoners, and then among the ship's company, and on the 29th a case of cholera occurred. There was nothing peculiar in the atmosphere or in the condition of the ship, but it was not until the Russians were received that there were any indications of stomach or bowel complaints. During the passage, while in the Great Belt, the ship came into juxtaposition with the *St. Vincent,* in which vessel the disease was rife and on the increase on that day; on that day, too, there was an increase of diarrhœa in the *Algiers,* which was towing the *St. Vincent.* On the following day she was cast off, and the ships separated, when the diarrhœa immediately decreased. It should be observed, however, that at the same time a gale of wind from the southward set in, after which all diarrhœa subsided. The chief predisposing and exciting causes of the disease seemed to be exposure and fatigue, with irregularity and sudden change of diet. That the ship suffered so little might be attributed to the superior character of the crew, who were seasoned sailors, and had orderly and cleanly habits. The only remedies used were at first, opium two grains and calomel ten grains, and these means were repeated in diminished doses. Externally, hot water in tins was applied to the scrobiculus cordis, spine, and extremities; and to allay cramps, strong friction was applied. The preventive measures recommended by Mr. Andrews are, that the ship should be kept as dry and clean and as well ventilated as possible; that all offensive odours emanating from the holds, bilges, pump-well, etc., should be destroyed by a plentiful distribution of the chloride of zinc; that the men should be kept personally clean, that their hammocks and bedding should be frequently exposed to the sun and air, that they should not be needlessly exposed or harassed with excessive exercises, that their comforts should be attended to, and contentment and cheerfulness created and fostered; that only wholesome fruits and vegetables and in fresh condition should be allowed on board; that all deviations from health, especially diarrhœa, should be promptly attended to and treated; that the leading men should be made aware of the necessity of watching the rest and reporting their state to the medical officer; and, lastly, that no man coming from a choleraic ship or locality should be placed on board a clean ship.

It only remains that we should give a summary of the preceding observations.

First, the question with regard to *predisposing causes* is answered by the medical men of fifteen ships only, and their opinions may all be enumerated as follows:—exposure to sudden changes of heat, cold, moisture, and night air, bad food and clothing; intemperance; immoderate use of fruits and vegetables; drinking cold water, when hot; hard work, causing fatigue; crowding; bad ventilation; the use of impure water; finally, diarrhœa as a consequence of the foregoing causes, either singly or in combination.

With respect to the *exciting cause*, the question is answered by the medical men of sixteen ships only; of these, ten consider it to have been atmospheric influence; one considers the poison to have been atmospheric, but rendered contagious by evacuations from the sick, and by foul air; while the remaining five, without mentioning the term, evidently lean to the side of contagion.

The question regarding the *cause of comparative immunity* received answers from the medical men of sixteen ships, and may be thus comprised. The immunity is ascribed to shifting of locality; to cleanliness, dryness, and thorough ventilation of the ship; to keeping the men out of the sun and fully occupying them; in one case to the excellent health and regular habits of the men and their non-communication with the shore; to the free use of chloride of zinc; to the use of quinine wine as a preventive; to the avoidance of unripe fruit; and to the early treatment of diarrhœa. In one case it is made a query whether the use of distilled water may not have prevented the occurrence of the disease.

On the subject of *treatment*, we have answers from all those ships which were visited by either cholera or diarrhœa, but there were twelve which escaped altogether. It seems to have been a very frequent practice to commence with a large dose of calomel and opium, and then to give small doses at frequent intervals. In at least eighteen of the ships calomel seems to have been the leading medicine. Stimulants, both internal and external, were also much used. Dr. Billing's plan by tartar emetic is extolled by one gentleman, and Dr. Stevens's saline plan by another; but no such measure of success seems to have followed any mode of treatment as to recommend it emphatically beyond the rest. The only novelty that we call to mind is the application of cupping glasses over the limbs and body in large numbers, which is stated by Mr. O'Brien, of the *Wrangler*, to have had a good effect in restoring warmth and preventing cramp, but he had only one or two cases of cholera under treatment.

The last, and by no means the least important part of our summary, has reference to the suggestions offered with regard to *prophylaxis*, and they admit of division into those which respect the ship, and those which respect the men. As to the former, the chief recommendations are cleanliness, ventilation, fumigation, whitewashing, dryness, the free use of chloride of zinc, and, as the most important of all, a shifting of locality. For the sake of effecting a speedy drying of the ship, the use of hot water for washing decks is recommended by one gentleman, and swinging stoves by another. In one instance it is suggested that in steam ships the bilges should be lined with metal, to prevent the absorption of grease, and that they should be flushed with a hot alkaline solution previously to purification by chloride of zinc. In one instance, also, the closure of the scuttles and ports at night, is suggested.

The preventive measures recommended, as applicable to the men, are as follows:—A generous diet with fresh provisions when procurable; recreation; amusement; comfort; warm clothing; the use of flannel in general, and especially a belt round the abdomen; the use of quinine wine; a strict surveillance and immediate attention to diarrhœa, however slight; the serving out of tea or coffee before going on deck in the morning; a sufficient supply of clean bedding, well aired; the prevention of intercourse with any ship or locality where cholera exists; abstinence from unripe fruits; from excesses of all kinds; from exposure to night air, wet, and a hot sun; or to the early morning air without food; the avoidance of fatigue, especially in the sun, and of exposure to sudden chills, or long fasting.

Such, gentlemen, is the substance and such the summary of a report for which, when we consider the arduous duties which devolved upon the medical officers of our fleets, this society and the public at large ought to consider themselves greatly indebted. Some of the suggestions it contains are very valuable, and it offers much food for reflection on a disease, the nature of which still remains so obscure. It has necessarily extended to so great a length that I will only crave your indulgence just to make one or two observations, which I think important.

The most striking fact it contains is, in my opinion, the great disproportion between the liability to cholera of the officers and that of the men under their command. Out of 884 officers in the Black Sea, on board the ships mentioned in this report, there were but five who took the disease, and of these one was a gunner and one a boatswain, whose habits, probably, assimilated more to that of foremast men than of officers of the quarter-deck. This gives a proportion of 1 to 177: while in the case of the men who, exclusive of officers, amounted to 11,488, there were 705 attacks, or 1 in about 16·29.

In the Baltic, where there were in the seven ships, from which we have reports, 183 officers, there was not a single case of cholera among them; while among the men, who, exclusive of officers, amounted to 1841, there were 49 attacks, or 1 in 37·57.

Now, if we assume the exciting cause to have been, in both classes, the same, or nearly the same (and whether we look to atmospheric influence or an emanation fiom the bodies of men, we can scarcely refuse to admit this, since all were living almost promiscuously in the same vessel), we are forced to attribute the difference chiefly to the predisposing and, in a great measure, preventable causes, and thence to coincide with those who recommend as prophylactics, cleanliness, ventilation, good clothing, and diet (fresh provisions), temperance, moderation in exertion and amusement. Whether the spirit drinking of the men may predispose to the disease more than the wine drinking of the officers, is a question worthy of further investigation. There ought to be some discoverable cause for so vast a difference.

With regard to the proportion of deaths to attacks, the officers are in excess, for four out of the five cases died—but scarcely any reliable conclusion can be drawn from numbers so small. Among the men in the Black Sea the proportion of deaths to attacks is 1 to 1·8, and in the Baltic 1 to 2·88; but we must bear in mind that the report from the latter is very imperfect, comprising only seven ships altogether, four of which had no cholera. Comparing the sailors, whose duties differ considerably from those of the marines, with this latter body, we find that the proportion of the latter to the former is, in the Black Sea, 2,353 to 8,945, or 1 to 3·8.

The number of marines attacked in the Black Sea was 193, which, their numbers being 2,353, gives a proportion of 1 to 12·19; whereas the sailors, as stated above, were attacked in the proportion of only 1 to 16·29.

The proportion of deaths to attacks among the marines in the Black Sea was as 1 to 1·6. The disease was, therefore, a little more fatal, and notably more frequent among them, than among the sailors. In the Baltic the number of the marines was 384, and that of the sailors 1,448, or 1 marine to 3·77 sailors. The number of attacks among the marines was nine, or 1 in 42·6; and there was only one death.

It would have been very desirable to have been able to institute a similar comparison between the engineers and stokers, and the men otherwise employed: but unfortunately in by far the majority of the reports, they have not been separated from the other sailors.

I have already given the facts respecting premonitory symptoms, which are much at variance with the notions of some, but are yet so distinctly stated, especially by the medical officer of the *Britannia,* who, from his manner of answering, seems evidently to have been fully aware of the import of the question, that we can scarcely doubt their correctness, if not in all, at least in many of the cases.

Of the suggestions as to preventive measures, I consider by far the most valuable that which recommends a shifting of locality. It seems to have proved successful in several instances, and those who consider the exciting cause of cholera to exist in the atmosphere, will regard this as a circumstance strongly favouring their belief.

Gentlemen, I beg, in conclusion, to apologise for having occupied so much of your time; but I felt that unless such materials as have been courteously furnished at our request, were treated in full detail, justice could not be done either to them, or to those meritorious officers from whom they have been obtained.

